# Should Lung-Sparing Surgery Be the Standard Procedure for Malignant Pleural Mesothelioma?

**DOI:** 10.3390/jcm9072153

**Published:** 2020-07-08

**Authors:** Yoshinobu Ichiki, Hidenori Goto, Takashi Fukuyama, Kozo Nakanishi

**Affiliations:** 1 Department of General Thoracic Surgery, National Hospital Organization, Saitama Hospital, 2-1 Suwa, Wako, Saitama 351-0102, Japan; goto.hidenori.sv@mail.hosp.go.jp (H.G.); nakanishi.kozo.tf@mail.hosp.go.jp (K.N.); 2 Second Department of Surgery, School of Medicine, University of Occupational and Environmental Health, Kitakyushu 807-8555, Japan; 3 Division of Biomedical Research, Kitasato University Medical Center, Kitamoto 364-8501, Japan; fukuyam@insti.kitasato-u.ac.jp

**Keywords:** mesothelioma, pulmonary complication, pleurectomy, decortication, extrapleural pneumonectomy

## Abstract

Background: Surgical procedures for malignant pleural mesothelioma (MPM) include extrapleural pneumonectomy (EPP), extended pleurectomy/decortication (P/D) and P/D. EPP has been applied to MPM for a long time, but the postoperative status is extremely poor due to the loss of one whole lung. We compared the mortality, morbidity and median survival time (MST) of lung-sparing surgery (extended P/D or P/D) and lung-sacrificing surgery (EPP) for MPM by performing a systematic review. Methods: We extracted the number of events and patients from the literature identified in electronic databases. Ultimately, 15 reports were selected, and 2674 MPM patients, including 1434 patients undergoing EPP and 1240 patients undergoing extended P/D or P/D, were analyzed. Results: Our systematic review showed that lung-sparing surgery was significantly superior to lung-sacrificing surgery in both the surgical-related mortality (extended P/D vs. EPP: 3.19% vs. 7.65%, *p* < 0.01; P/D vs. EPP: 1.85% vs. 7.34%, *p* < 0.01) and morbidity (extended P/D vs. EPP: 35.7% vs. 60.0%, *p* < 0.01; P/D vs. EPP: 9.52% vs. 20.89%, *p* < 0.01). Lung-sparing surgery was not inferior to EPP in terms of MST. Conclusion: Although no prospective randomized controlled trial has been conducted, it may be time to change the standard surgical method for MPM from lung-sacrificing surgery to lung-sparing surgery.

## 1. Introduction

**Figure 1:**
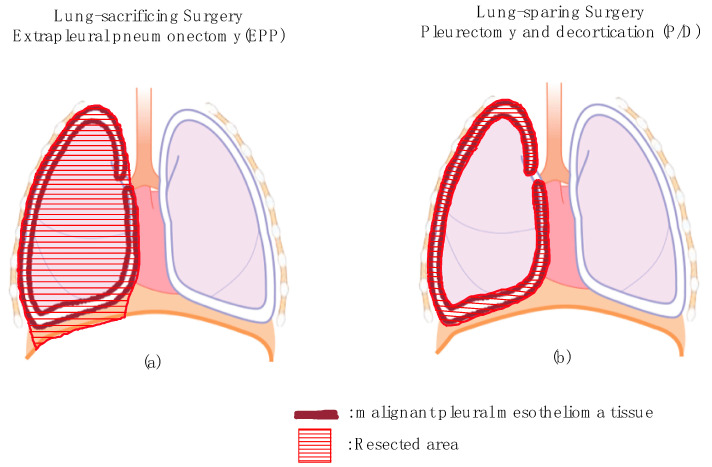
Surgical procedure for malignant pleural mesothelioma (MPM). (**a**) Lung-sacrificing surgery (extrapleural pneumonectomy, EPP) is shown. After the ipsilateral parietal pleura, lung, diaphragm and pericardium are removed, reconstruction of the diaphragm and pericardium is performed. (**b**) Lung-sparing surgery (pleurectomy/decortication, P/D) is shown. The ipsilateral parietal and visceral pleura are removed.

Malignant pleural mesothelioma (MPM) is a rare and aggressive malignancy with a poor prognosis. MPM is closely related to asbestos exposure and occurs long after the exposure. Opportunities for asbestos exposure were related to its history of widespread use in construction and insulation projects. Surgery plays the most important role in treating MPM. Microscopic complete resection (R0) is difficult due to the remaining tumor exfoliation surface, so the aim of the surgery is to perform macroscopic complete resection (R1). Combination therapy using chemotherapy and radiotherapy is also important for controlling microscopic remaining tumor cells. There are two main types of surgery performed in such cases: lung-sacrificing surgery and lung-sparing surgery (Figure 1). 

Lung-sacrificing surgery is extrapleural pneumonectomy (EPP). The ipsilateral parietal pleura, lung, diaphragm and pericardium are removed en bloc, and then reconstruction of the diaphragm and pericardium is performed using an artificial seat. Lung-sparing surgery is pleurectomy/decortication (P/D) and extended P/D. P/D indicates removal of the parietal and visceral pleura while preserving the diaphragm and pericardium. Extended P/D is a procedure involving the removal of not only the parietal and visceral pleura but also the diaphragm and pericardium, followed by reconstruction of the diaphragm and pericardium.

1. EPP: en bloc resection of the visceral and parietal pleura, lung, ipsilateral hemidiaphragm and pericardium.
2. P/D: parietal and visceral pleurectomy.
3. Extended P/D: parietal and visceral pleurectomy with resection of diaphragm and pericardium.

P/D, which was originally used to manage chronic empyema [1,2], has been applied to MPM in recent years. Sarot et al. reported the EPP technique for tuberculous infection resistant to collapse therapy or thoracoplasty in 1949 [3]. Since Butchart et al. demonstrated the utility of EPP for managing MPM patients in 1976 [4], EPP has been the standard surgical procedure. The advantages and disadvantages associated with each procedure are shown in Table 1. 

Advantages of lung-sacrificing surgery include fewer microscopic residual tumor cells, a tendency toward a shorter operation, less air leakage and easier performance of adjuvant radiation than with lung-sparing surgery [5]. Since the entire diseased lung is removed, the number of microscopic residual tumor cells can be reduced, and air leakage from the lung is not observed. However, lung-sacrificing surgery is a more invasive procedure than lung-sparing surgery. It is also more difficult to receive adjuvant chemotherapy than with lung-sparing surgery due to the patient’s worse postoperative physical condition. 

Advantages of lung-sparing surgery include better safety and a better quality of life (QOL), less need for extensive reconstruction and a higher ability to tolerate more aggressive chemotherapy than the lung-sacrificing surgery [6]. Recently, immune checkpoint inhibitors have been applied to MPM patients. We previously reported powerful immune reactions against tumor cells caused by cytotoxic T lymphocytes and B lymphocytes in the lung [7,8,9]. Theoretically, a lung-derived tumor immune response may be left by preserving a lung with lung-sparing surgery. However, this approach requires a skilled technique and the operation time of lung-sparing surgery tends to be long. 

While EPP has been the standard procedure for MPM for a long time, good postoperative outcomes with P/D have been reported in recent years. Which approach is better from the viewpoint of surgical safety and tumor curability? Unfortunately, no randomized trial comparing these techniques has yet been conducted. We hypothesized that lung-sparing surgery should be selected for all MPM patients, and that all surgeons performing surgery for MPM may need to develop skills for lung-sparing surgery. 

We herein report our comparison of these techniques and consider each surgical approach for MPM by performing a systematic review using a large number of studies with regard to the mortality, morbidity and median survival time (MST) after surgery. 

## 2. Methods

### 2.1. Search Strategy and Article Selection

A literature search was performed in PubMed using the following terms “mesothelioma”, “malignant pleural mesothelioma”, “extrapleural pneumonectomy”, “EPP”, “pleurectomy”, “decortication”, “P/D” and “extended P/D”. The selection criteria of this review are as follows: (1) ≥10 patients involved, (2) English papers, (3) reports regarding the comparison of surgical methods for MPM patients, (4) human subjects and (5) papers published from 1990 to 2020. Duplicate articles, abstracts, case reports, conference presentations, editorials and expert opinions were excluded. Review articles were also excluded to avoid publication bias. We extracted the numbers of events and patients from the articles’ text, tables and figures. There were several papers that described extended P/D and P/D as the same method. Therefore, if the text mentioned resection and reconstruction of the diaphragm and pericardium, it was regarded as extended P/D, even if it was described as P/D in the paper.

### 2.2. Statistical Analyses

A meta-analysis of the postoperative complications and mortality data obtained from the papers was performed. The Odds Ratio (OR) was used as a summary statistic. X^2^ tests were applied to evaluate heterogeneity. I^2^ was calculated as: 100% × (Q-df)/Q, with Q defined as Cochrane’s heterogeneity statistic and df defined as the degrees of freedom [10]. If I^2^ was more than 50%, heterogeneity was considered to be present. All *p*-values were two-sided. When the *p*-value was less than 0.05, we regarded the difference as significant. 

Statistical analyses were performed with EZR (Saitama Medical Center, Jichi Medical University, Saitama, Japan), which is a graphical user interface for R (The R Foundation for Statistical Computing, Vienna, Austria). More precisely, it is a modified version of R commander designed to add statistical functions frequently used in biostatistics [11]. 

## 3. Results

**Figure 2:**
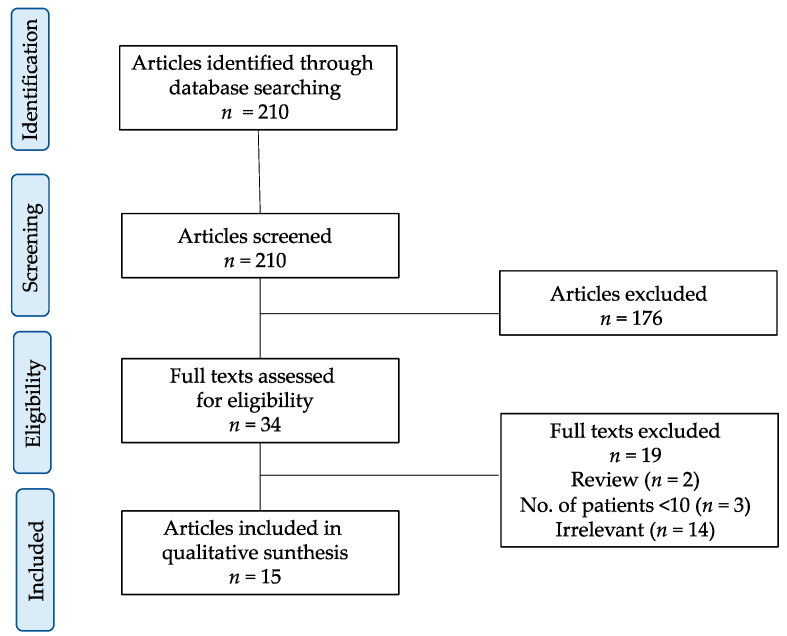
Search strategy for the meta-analysis for “extended P/D vs. EPP” and “P/D vs. EPP”.

A total of 210 papers were identified by a literature search. Irrelevant papers were excluded, and 34 potentially relevant papers were selected according to the selection criteria. Ultimately, 15 papers that met the selection criteria were selected [12,13,14,15,16,17,18,19,20,21,22,23,24,25,26]. A total of 2674 MPM patients, including 1434 patients undergoing EPP and 1240 undergoing extended P/D or P/D, were analyzed in this study (Figure 2). 

### 3.1. Extended PD vs. EPP

**Figure 3:**
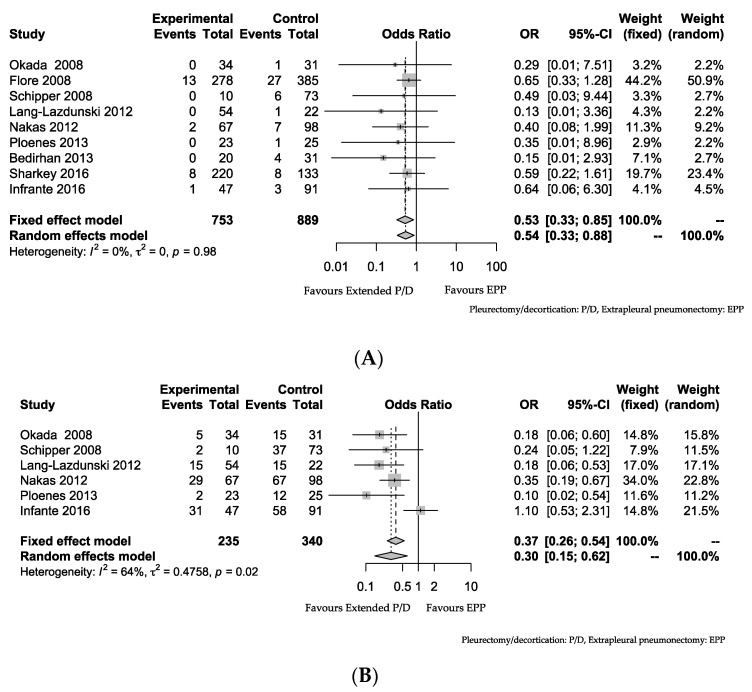
(**A**) Comparison of postoperative mortalities of Extended P/D and EPP. Forest plot of the odds ratio (OR) of the mortality after extended pleurectomy/decortication (P/D) vs. extrapleural pneumonectomy (EPP) in surgery for malignant pleural mesothelioma (MPM). (**B**) Comparison of postoperative morbidities of Extended P/D and EPP. Forest plot of the OR of the morbidity after extended P/D vs. EPP in surgery for MPM. The estimate of the OR of each study corresponds to the middle of the square, and the horizontal line shows the 95% confidence interval (CI). On each line, the event number as a fraction of the total number treated is shown for both treatment groups. For each subgroup, the sum of the statistics, along with the summary OR, is represented by a solid diamond. The results of a test of heterogeneity between the trials within a subgroup are given below the summary statistics.

The 30-day mortality was significantly higher in patients receiving EPP than in those receiving extended P/D according to the meta-analysis (OR: 0.53 (95% confidence interval (CI): 0.33, 0.85); *p* < 0.01, I^2^ = 0%). The 30-day mortality rates of extended P/D and EPP were 3.19% and 7.65%, respectively (*p* < 0.01). Although the morbidity was also significantly higher in patients receiving EPP than in those receiving extended P/D (OR: 0.37 (95% CI: 0.26, 0.54); *p* < 0.01, I^2^ = 64%), heterogeneity was detected (Figure 3). One report found that the morbidity of patients receiving EPP was similar to that of patients receiving extended P/D [17]. The morbidity rates of extended P/D and EPP were 35.7% and 60.0%, respectively (*p* < 0.01). Five of the seven papers reported a tendency toward a longer MST for extended P/D than for EPP [10,12,13,16,18]. Only one article found that extended P/D had a significantly longer MST than EPP; in the other six papers, there was no significant difference in the MST between extended P/D and EPP (Table 2). 

### 3.2. PD vs. EPP

**Figure 4:**
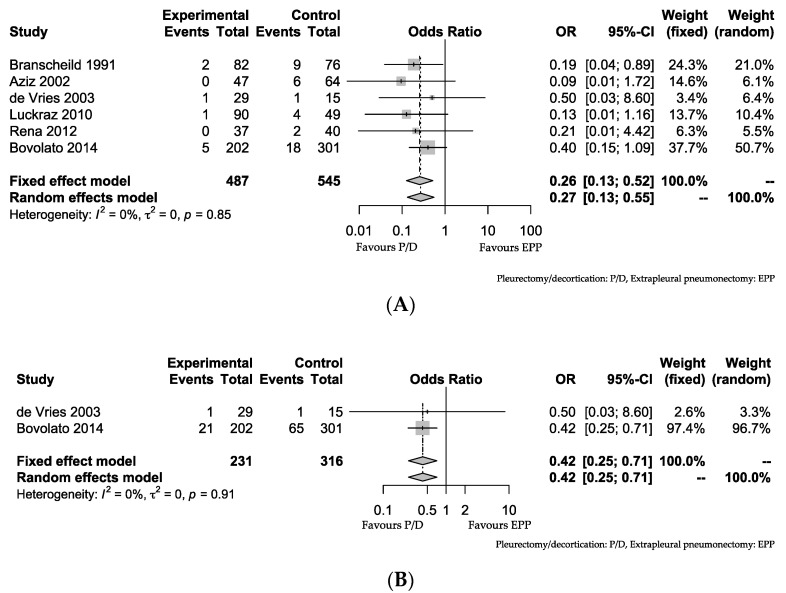
(**A**) Comparison of postoperative mortalities of P/D and EPP. Forest plot of the odds ratio (OR) of the mortality after pleurectomy/decortication (P/D) vs. extrapleural pneumonectomy (EPP) in surgery for malignant pleural mesothelioma (MPM). (**B**) Comparison of postoperative morbidities of P/D and EPP. Forest plot of the OR of the morbidity after P/D vs. EPP in surgery for MPM. The estimate of the OR of each study corresponds to the middle of the square, and the horizontal line shows the 95% confidence interval (CI). On each line, the event number as a fraction of the total number treated is shown for both treatment groups. For each subgroup, the sum of the statistics, along with the summary OR, is represented by a solid diamond. The result of a test of heterogeneity between the trials within a subgroup are given below the summary statistics.

The 30-day mortality was significantly higher in patients receiving EPP than in those receiving P/D according to a meta-analysis (OR: 0.26 (95% CI: 0.13, 0.52); *p* < 0.01, I^2^ = 0%). The 30-day mortality rates of P/D and EPP were 1.85% and 7.34%, respectively (*p* < 0.01). The morbidity was also significantly higher in patients receiving EPP than in those receiving P/D (OR: 0.42 (95% CI: 0.25, 0.71); *p* < 0.01, I^2^ = 0%) (Figure 4). In four of the eight papers, EPP had a longer MST than P/D [16,18,23,24]. The morbidity rates of P/D and EPP were 9.52% and 20.89%, respectively (*p* < 0.01). There were no significant differences in the MST among the eight papers (Table 3).

## 4. Discussion

Dunn et al. revealed that operative death due to pneumonectomy for lung cancer was observed in 1 of 25 (4%) patients. The mean loss of forced vital capacity (FVC) and forced expiratory volume in 1 s (FEV_1.0_) were 41.5% and 40.2% after right pneumonectomy and 34.0% and 38.3% after left pneumonectomy, respectively [27]. We reported that the mortality and morbidity rates of pneumonectomy patients with lung cancer were 4.2% (3/71 patients) and 31.3% (22/71 patients), respectively. The causes of deaths were acute respiratory distress syndrome (ARDS) (2.8%) and interstitial pneumonitis (IP) (1.4%) [28]. The complications of pneumonectomy were arrythmia (21.2%), ARDS (7.0%), empyema (2.8%), bronchopleural fistula (2.8%), congestive heart failure (1.4%) and IP (1.4%). The postpneumonectomy status is unstable, and the reserved capacity is markedly decreased due to the loss of a whole lung, including the vascular bed, which reduces the cardiorespiratory function to about half of its baseline level. Since pneumonectomy is an extremely high-risk operation in lung cancer surgery, it should be avoided by instead performing bronchoplasty and angioplasty as often as possible.

EPP negatively influences the patient’s general status because of the post-pneumonectomy condition. EPP is a more invasive surgical method than pneumonectomy because pleurectomy and decortication in addition to pneumonectomy markedly increase the surgical invasion. Sugarbaker et al. reported complications after 328 cases of EPPs [29]. Postoperative complications, including minor ones, were observed in 198 of 328 (60.4%) patients and included atrial fibrillation (44.2%), extension of intubation period (7.9%), vocal cord paralysis (6.7%), deep vein thrombosis (6.4%), technical complications (patch dehiscence, hemorrhaging; 6.1%), tamponade (3.6%), ARDS (3.6%), cardiac arrest (3%), constrictive physiology (2.7%), aspiration (2.7%), renal failure (2.7%), empyema (2.4%), tracheostomy (1.8%), myocardial infarction (1.5%), pulmonary embolus (1.5%) and bronchopleural fistula (0.6%). There may be serious cardiopulmonary complications, and careful perioperative observation and treatment are required. 

Vocal cord paralysis often causes aspiration pneumonia. After pneumonectomy, the remaining lung is the dependent lung, so aspiration pneumonia after EPP is lethal. Furthermore, mediastinal deviation after EPP causes sputum retention which is a risk factor for postoperative pneumonia. Management of a thoracic drain, such as with non-continuous negative-pressure drainage, is important for controlling mediastinal deviation. Excreting sputum via bronchoscopy is an extremely useful treatment for preventing postoperative lethal pneumonia. In addition, bronchoscopy also provides information about not only the bronchus but also the vocal cord movement. 

Yan et al. analyzed the prognostic data of 70 MPM patients with EPP and revealed that the mortality and morbidity rates of EPP for MPM were 5.7% and 37%, respectively [30]. EPP is highly invasive and risky, so the management of postoperative complications is very important. The MARS trial was the first to randomly allocate MPM patients with induction platina-based chemotherapy to receive EPP with postoperative hemithoracic radiation (24 patients) or non-surgical therapy (26 patients). The MST of EPP and non-surgical therapy was 14.4 months and 19.5 months, respectively (hazard ratio (HR): 2.75; 95% CI: 1.21, 6.26; *p* = 0.016). Ten and two severe adverse events were observed in EPP and non-surgical patients, respectively. The trial showed that EPP within trimodal treatment brought no marked benefits and likely even harmed the patient, even based on limited data [31]. Theoretically, EPP is more radical and more invasive than P/D, so the management of perioperative complications, especially cardiopulmonary complications, is extremely important through early detection and treatment using computed tomography (CT) and bronchoscopy. 

P/D has attracted attention as a less invasive lung-sparing surgery than EPP. Lung-sparing surgery has benefits of preserving the cardiopulmonary function and improving the patient’s tolerance of aggressive postoperative treatments, even though more residual tumor cells may be left in the lung. The diaphragm is the most significant respiratory muscle. Although it is unclear how much function remains in the injured phrenic nerve and diaphragm, a greater pulmonary function, which can reduce postoperative complications, is theoretically preserved by sparing the lung than by sacrificing it. Chronic pleural empyema (CPE) is the final stage of pleural empyema. Since the pulmonary function of CPE patients is extremely reduced due to fluid collection in the chest cavity and atelectatic lung, P/D was previously used to the improve pulmonary function for CPE patients. Gokce et al. reported that P/D for CPE significantly improved the pulmonary function, going from an FEV_1.0_% of 61.4% and FVC% of 60.8% to FEV_1.0_% of 78.9% and FVC% of 77.4%, as shown in Table 4 [1]. It was reported that the chest wall deformity also improved after surgery because of re-expansion of the lung and enlargement of the intercostal spaces [1]. Ryman et al. also revealed that the spirometry data were significantly improved after P/D for CPE, going from FEV_1.0_% of 50.0% and VC% of 62.3% to FEV_1.0_% of 69.2% and VC% of 79.8% [2]. Since P/D for benign disease can preserve the phrenic nerve and diaphragm, a significant improvement in the pulmonary function is expected. Unilateral diaphragm dysfunction brought about a reduction in the pulmonary function (FEV_1.0_%: −30% and FVC%: −25%) in healthy patients [2]. Bölükbas et al. reported that P/D for MPM significantly improved the pulmonary function at 2 months after surgery, going from FEV_1.0_% of 60.2% and FVC% of 54.7% to FEV_1.0_% of 73.6% and FVC% of 68.9% [32]. Furthermore, a greater improvement in the respiratory function was observed in cases with a preserved diaphragm function than in those which lost a diaphragm function with an FEV_1.0_% of +17.1% and FVC% of +23.1% after surgery. Extended P/D for MPM did not improve the overall health-related QOL and actually showed a negative influence on the pulmonary function in performance status (PS) 0 patients [33]. The preoperative pulmonary function of PS 0 patients (FEV_1.0_%: 84.1% and FVC%: 80.2%) significantly decreased postoperatively (FEV_1.0_%: 72.4% and FVC%: 67.1%). The postoperative pulmonary function of PS 1 and 2 patients was not significantly different from the preoperative status (FEV_1.0_%: 65.6% to 72.8% and FVC%: 61.7% to 64.8%). Extended P/D for MPM significantly reduced the pulmonary function in patients with few symptoms. However, Tanaka et al. reported that P/D for MPM reduced the pulmonary function after surgery (FEV_1.0_: 2.65 L to 2.00 L, and FVC: 3.53 L to 2.51 L) [34]. Each analysis was performed in a limited number of cases, so the results seemed to differ, even in cases treated with the same surgical method for the same disease. Ploenes et al. compared the postoperative pulmonary function of P/D with that of EPP [17]. Although the postoperative pulmonary function after P/D for MPM was not significantly decreased compared with before surgery (median FEV_1.0_%: 87.1% to 70.0% and FVC%: 86.5% to 68.2%), the postoperative pulmonary function after EPP for MPM was significantly decreased (median FEV_1.0_%: 78.0% to 49.3% and FVC%: 76.8% to 48.0%). They concluded that P/D was able to preserve the postoperative pulmonary function, while EPP significantly decreased the pulmonary function after surgery, which might result in increased pulmonary complications and a reduced QOL. Although there has been no prospective randomized controlled trial focusing on surgical methods for MPM, P/D seems to be superior to EPP in terms of the postoperative pulmonary function. 

Magouliotis et al. also reported a meta-analysis of the data of 1672 MPM patients undergoing EPP and 2236 MPM patients undergoing P/D, including extended P/D [35]. P/D showed a significantly better median overall survival and 30-day postoperative mortality than EPP. EPP, by contrast, caused significantly more postoperative complications, such as atrial fibrillation, hemorrhaging, empyema and bronchopleural fistula. Based on these findings, it was concluded that P/D was a better surgical method procedure for MPM when feasible. Cao et al. performed a systematic review to compare outcomes of EPP and extended P/D. The data on 632 MPM patients undergoing EPP and 513 MPM patients undergoing extended P/D was analyzed, and comparison of these two groups demonstrated significantly lower perioperative mortality (2.9% vs. 6.8%, *p* = 0.02) and morbidity (27.9% vs. 62.0%, *p* < 0.0001) for patients undergoing extended P/D than EPP [36]. Taioli et al. examined and performed a meta-analysis of the data of 1512 MPM patients undergoing P/D and 1391 MPM patients undergoing EPP. There were significantly more short-term (within 30 days after surgery) deaths in the patients undergoing EPP than P/D (percent mortality meta estimate: 4.5% vs. 1.7%, *p* < 0.05) [37]. 

Schwartz et al. reviewed and compared the postoperative quality of life (QOL) of P/D and EPP [38]. They measured the QOL using the European Organization for Research and Treatment of Cancer (EORTC) QLQ-C30 and concluded that patients receiving P/D had a higher postoperative QOL than those receiving EPP, although at a limited level of evidence. We also reported that an MPM patient receiving P/D and postoperative chemotherapy (cisplatin and pemetrexed) was able to return to work. Furthermore, the patient has been working continuously for more than six years without recurrence [39]. The patient could balance treatment for MPM and work, earn her income and contribute to socio-economic activities. Since P/D can maintain a relatively high QOL after the surgery, its application is expanding to diseases other than MPM. We applied extended P/D to an operation for thymoma with pleural dissemination. There were no serious adverse events postoperatively, and the patient has been living without recurrence for more than one year since the surgery [40]. Although it needs to be clarified by a prospective randomized controlled trial, extended P/D may become a good indication for thymoma with pleural dissemination. 

Given these previous reports, while we have performed EPP as a standard surgical method for MPM for a long time, in reality, lung-sparing surgeries, such as P/D or extended P/D, are superior in terms of safety. Since no randomized controlled trial for comparing the surgical methods for MPM has been performed, we performed the present study. In our review, we performed a meta-analysis to evaluate the best surgical method for MPM. Both the 30-day postoperative mortality and morbidity were significantly higher for EPP than for P/D or extended P/D. The 30-day mortality rates of extended P/D vs. EPP and P/D vs. EPP were 3.19% vs. 7.65% (*p* < 0.01) and 1.85% vs. 7.34% (*p* < 0.01), respectively. The morbidities of extended P/D vs. EPP and P/D vs. EPP were 35.7% vs. 60.0% (*p* < 0.01) and 9.52% vs. 20.89% (*p* < 0.01), respectively. In addition, lung-sparing surgery was not inferior to EPP in terms of the MST. These findings suggest that P/D or extended P/D should be performed to reduce surgical-related mortality and morbidity and obtain a sufficiently good prognosis if technical and macroscopic complete resection is possible. 

However, each report used for this meta-analysis has its own primary endpoint that differs from that of other studies. The criteria of perioperative complications also differ among institutes. Heterogeneity among the morbidity data of extended P/D vs. EPP was detected in our meta-analysis. Although the morbidity was also significantly higher in patients receiving EPP than in those receiving extended P/D, a literature search revealed that the morbidity of patients receiving EPP was similar to that of patients receiving extended P/D [20]. There were some papers in which the surgical procedure was described as P/D even though extended P/D was actually performed. Some papers seem to have used a broad definition of P/D that included extended P/D. We judged whether the surgery was P/D or extended P/D after confirming all of the details of the surgical method in the article texts. However, there were a few papers that lacked a clear description of the surgical method, which was a limitation of this analysis. Only two papers were available for comparing the morbidity of P/D and EPP. Since all of the papers used for this meta-analysis covered retrospective studies and did not include prospective randomized controlled trials, we used the OR rather than the risk ratio to perform our systematic review. It is thought that the surgical indication factors such as histological type, TNM staging and patient characteristics (age, cardiovascular and pulmonary risk factors) are different in each institution, and the quality of surgery is also different, but such information was not included in this analysis. There is a possibility that it is a limitation. 

The current staging system for MPM was developed in 1994 at a workshop sponsored by the International Association for the Study of Lung Cancer (IASLC) and the International Mesothelioma Interest Group (IMIG), during which MPM investigators analyzed reported surgical databases and the available small clinical trials on MPM. The resulting TNM-based system had potential applicants in the clinical, surgical and pathologic staging of MPM [41]. Then, the Union for International Cancer Control (UICC) and the American Joint Commission on Cancer (AJCC) accepted it as the first international MPM staging system for the sixth edition of their staging manuals, and now the eighth edition of the UICC/AJCC is used for staging of MPM [42]. TNM staging is important in determining treatment strategy and predicting individual prognosis. The T component should provide accurate prognostic information, and survival rate should decrease as T factor increases. T component should provide information so that evidence-based treatment options can be selected. Nowak et al. performed T-component analysis using the data of 509 cases that had only clinical staging, 836 cases that had only surgical staging, and 642 cases that had both available, and proposed to combine clinical and pathological T1a (involvement of the ipsilateral parietal pleura) and T1b (involvement of the ipsilateral visceral pleura) in the seventh edition of the UICC/AJCC into a single T1 category in the eighth edition. It is difficult to distinguish the invasion of the parietal pleura and the invasion of the visceral pleura only by clinical information. Furthermore, surprisingly, pathological T1a and T1b in the seventh edition could not be accurately distinguished [43]. A prognosis of involvement of the contralateral pleura or transdiaphragmatic extension of tumor to the peritoneum was as unfavorable as formation of distant metastasis [44]. Aziz et al. showed that T1 and T2 patients had a particularly good survival outcome after the surgery, therefore T stage was carefully evaluated to determine the indication of surgery [22]. There is also a possibility that surgical indications based on TNM staging differ among each institute.

It was reported that the negative significance of lymph node involvement on a prognosis after EPP was observed. The bad prognosis of lymph node involvement in the survival of MPM following surgery and chemotherapy was not supported [45]. On the other hand, Faber et al. reported that a worse prognosis in patients with lymph node involvement was not observed [46]. The treatment strategy is also controversial for MPM with lymph node involvement. Rice et al. investigated the relationship between lymph node metastasis and prognosis using the data of 2432 MPM patients. Nodal metastases were clinically observed in only 22% of MPM in 1029 patients, whereas 321 of 851 (38%) patients receiving an operation had nodal metastases as a surgical finding [47]. Actually, it often is difficult to clinically identify nodal metastasis using computed tomography (CT) and positron emission tomography (PET). There was no significant difference of prognosis between metastases of nodes within the pleural reflection (hilar and parenchymal station, stations 10–14) and those of nodes in ipsilateral mediastinal nodes outside the pleural reflection (station 2–9). Therefore, any ipsilateral, intrathoracic node with metastatic involvement are defined as N1, and ipsilateral supraclavicular or contralateral nodes are defined as N2 [42]. 

MPM was distinguished into principal histologic subtypes according to the WHO 2004 classification of tumors of the pleura [48]: the epithelioid, the sarcomatoid, the biphasic (a mixture of both including at least 10% of each growth pattern) and desmoplastic subtype. Then, the same classification of MPM was carried over to the 2015 WHO [49,50]. On the other hand, we have to distinguish diffuse malignant mesothelioma (DMPM) from other mesothelioma which have a much better prognosis, including localized malignant mesotheliomas (LMPMs) and well-differentiated papillary mesotheliomas (WDPMs). As epithelioid mesothelioma is already known to have a better prognosis, lung-sacrificing surgery and lung-sparing surgery are preferred in patients with epithelioid MPM [51,52,53,54,55]. Edwards et al. identified non-epithelioid histological subtype, poor performance status, low hemoglobin, male gender, high platelet count, high lactate dehydrogenase and high white blood cell count as poor prognostic factors [52]. Furthermore, tumors with anaplastic or prominent giant cells, often multinucleated, are designated pleomorphic. It was reported that the pleomorphic subtype of epithelioid DMPM had a significantly poorer prognosis than other epithelioid MPM, and survival of pleomorphic subtype was similar to that of patients with sarcomatoid and biphasic DMPMs [56,57]. Although there was suggestion to make a new classification that changes pleomorphic pattern from the epithelioid to sarcomatoid subtype, this was not accepted by the WHO panel. These tumors are categorized as a poor prognostic subset of epithelioid DMPMs according to the 2015 WHO classification. There are also rare histologic subtypes of malignant pleural tumors that we previously reported. We reported a case of a patient with pleural epithelioid hemangioendothelioma (EHE), who received EPP. The prognosis was poor due to high malignant potential of pleural EHE [58]. Bias of histology may affect the overall prognosis because detailed analysis of histologic subtypes was not included in this study. 

There may be a bias of the ability of patients to receive adjuvant therapy based on their postoperative status, which is related to the differences in survival between patients receiving multimodality therapy versus those receiving surgery alone. The large study of 1365 consecutive MPM patients demonstrated that the patients with good prognosis factors (age < 70 years, epithelioid histological subtype) statistically showed a similar survival whether they received medical therapy only (*n* = 172), P/D (*n* = 202) or EPP (*n* = 301). However, patients receiving palliative treatment or chemotherapy alone had overall median survival of 11.7 months (95% CI: 10.5–12.5 months) in comparison to those receiving P/D with a median survival of 20.5 months (95% CI: 18.2–23.1 months), which shows a clear tendency to bring a 9-month survival benefit in favor of surgery. Median survival of patients receiving surgical resection with adjuvant therapy was significantly longer than that of patients receiving chemotherapy only (19.8 vs. 11.7 months; χ^2^_2df_ = 74.541; *p* < 0.0001) [23]. However, it included the data from almost 30 years ago. It is necessary to consider the situation where anti-cancer drugs were not yet developed as they are today.

A new feasibility study (MARS 2) is recruiting in the United Kingdom (MARS 2: A Feasibility Study Comparing (Extended) Pleurectomy Decortication Versus no Pleurectomy Decortication in Patients with Malignant Mesothelioma; ClinicalTrials.gov Identifier: NCT0240272). After 2 cycles of induction chemotherapy with cisplatin/pemetrexed, patients will be randomized to receive only (4 cycles of cisplatin/pemetrexed) or lung-sparing surgery plus chemotherapy (4 cycles of cisplatin/pemetrexed). The primary endpoint of MARS 2 is the ability to randomize 50 patients within the first 24 months or the ability to recruit 25 patients within any 6-month period. This study will bring us valuable information about lung-sparing surgery in the near future.

Immune checkpoint inhibitors (ICIs), such as anti-programmed cell death protein l (PD-1) antibody, has been applied to treatment of advanced MPM [59]. ICIs have become one of the important drug therapies for MPM in clinical practice. Alley et al. reported that 25 patients with programmed cell death-ligand 1 (PD-L1)-positive MPM received ICI (anti-PD-1 antibody), and 20% of the patients receiving ICI had a partial response and 52% had stable disease with a durable response time of 12 months [59]. We reported that there were effective immune responses against lung cancer in the lung [7,8,9] and introduced the excellent effects of the latest combination therapy of immunotherapy and chemotherapy [60]. We also identified 4 tumor antigens recognized by tumor infiltrating B cells in an MPM patient and clarified the existence of MPM-specific humoral immune response [61]. Additionally, we analyzed the clinicopathological data of 44 non-small cell lung cancer patients receiving ICI (anti-PD-1 antibody) and identified the Eastern Cooperative Oncology Group performance status (ECOG PS), pathological type, standardized uptake value (SUV) on PET, white blood cell count, neutrophil, neutrophil-to-lymphocyte ratio (NLR), lactate dehydrogenase and albumin as independent prognostic factors in multivariate analyses [62]. Similar considerations may be needed for more effective use of ICIs in MPM patients. Patil et al. analyzed 99 archival tumor tissues derived from advanced-stage MPM by performing an immunohistochemical assay of PD-L1 expression. Immune gene expression analysis of 87 of 99 archival tumor tissues was also performed by using NanoString analysis for 800 genes. PD-L1 expression was found in 16% of the MPM tumor samples, either in the tumor cells or the infiltrating immune cells. Finally, it was concluded that 60% of patients with MPM characterized by either PD-L1 expression or an inflamed status are attractive candidates for cancer immunotherapy [63]. Microsatellite instability (MSI) arises from inactivation of any of several mismatch repair (MMR) genes—mutL homolog 1 gene (*MLH1*), PMS1 homolog 2, mismatch repair system component gene (*PMS2*), mutS homolog 2 gene (*MSH2*), and mutS homolog 6 gene (*MSH6*)—resulting in failure to repair the routine errors which occur during replication of short repeats in a DNA sequence [64,65]. These translated proteins from heterodimers that repair DNA damage, with the common and relevant to tumorigenesis being mutL homolog 1 (MLH1)/PMS1 homolog 2, mismatch repair system component (PMS2) and mutS homolog 2 (MSH2)/mutS homolog 6 (MSH6) [66]. Confirming the status of MSI is now very important in predicting prognosis and selecting a treatment. The MSI-high status is thought to inactivate tumor suppressor genes, accumulate many genetic mutations and promote cancer development. It was reported that the MSI-high tumors showed a good response for ICI (anti-PD-1 antibody) because many gene mutations make tumors immunogenic [67]. Although Arulananda et al. performed a tissue microarray of tumor tissues of 335 patients with MPM to examine MSI status of MPM, they could not identify any patients with MPM with MSI [68]. On the other hand, it was reported that somatic mutations occurred in a number of tumor suppressor genes, such as cyclin-dependent kinase inhibitor 2A gene (CDKN2A), neurofibromin 2 (merlin) gene (NF2) and breast cancer susceptibility gene 1 (BRCA1)-associated protein 1 gene (BAP1). Additional gene alterations include several pathways (for example, the cell cycle, DNA repair, mitogen-activated protein kinase and phosphoinisitide-3-kinase/AKT pathways) [69,70,71]. Chimeric antigen receptor (CAR)-modified autologous T cells recognizing mesothelin (CART-meso cells) is also a new potential immunotherapy with growing evidence. Haas et al. reported that CART-meso cells were applied to 15 patients with chemotherapy refractory MPM, ovarian carcinoma and pancreatic ductal adenocarcinoma as a phase I study. Eleven of fifteen patients had stable disease with limited toxicity after a single infusion of CART-meso cells [72]. Immunotherapy is currently under development, and further excellent effects can be expected; therefore, lung-sparing surgery for MPM may become an even more important procedure. Multimodality therapy including intraoperative photodynamic therapy was also reported [73]. These promising treatments were applied to clinical practice to improve the survival of patients with MPM. 

Despite these limitations associated with our analysis and the lack of any randomized control trials with clear conclusions, we feel that we can recommend lung-sparing surgery, such as P/D or extended P/D, for treating MPM. Our results suggest that lung-sparing surgery is superior to EPP in terms of the surgery-related mortality and morbidity. It may be time to change the standard surgical method for MPM from lung-sacrificing surgery to lung-sparing surgery. 

## 5. Conclusions

This study is the first to compare the mortality, morbidity and MST separately for EPP vs. P/D and EPP vs. extended P/D. Our systematic reviews of EPP, extended P/D and P/D for MPM revealed that lung-sparing surgery (extended P/D or P/D) was superior to lung-sacrificing surgery (EPP) in terms of both the surgical-related mortality and morbidity. On the other hand, each study had different endpoints, different criteria for complications, different definitions of P/D and different surgical indications, such as TNM staging, histological type and patient characteristics, and this is a limitation in our study. However, surgeons who perform surgeries for MPM should study the techniques for lung-sparing surgery and perform lung-sparing surgery for MPM whenever possible, although there was no randomized controlled trial bringing clear supporting evidence.

## Figures and Tables

**Table 1:** Advantages and disadvantages of surgeries for MPM.

Advantage	Disadvantage
fewer microscopic residual tumor cells	worse safety and QOL *
a tendecy toward a shorter operation time	more need for extensive reconstruction
less air leakage	less ability to tolerate more aggressive
easier performance of adjuvant radiation	chemotherapy
better safety and QOL *	more microscopic residual tumor cells
less need for extensive reconstruction	a tendecy toward a longer operation time
a greater ability to tolerate more aggressive	more air leakage
chemotherapy preservation of immune reaction from the lung	more difficult performance of adjuvant radiation

*: quality of life.

**Table 2:** A summary of the median survival time of extended pleurectomy/decortication (P/D) vs. extrapleural pneumonectomy (EPP) for patients with malignant pleural mesothelioma (MPM).

Study	Extended P/D MST (Month)	Number of Patients Undergoing Extended P/D	EPP MST (Month)	Number of Patients Undergoing EPP
Okada 2008 [12]	17	34	13	31
Schipper 2008 [14]	17.2	10	16	73
Lang-Lazdunski 2012 [15]	23 *	54	12.8 *	22
Nakas 2012 [16]	13.4	67	14.7	98
Bedirhan 2013 [18]	27	20	17	31
Sharkey 2016 [19]	12.3	220	12.9	133
Infrante 2016 [20]	30	47	19	91

Pleurectomy/decortication: P/D, Extrapleural pneumonectomy: EPP, median survival time: MST *: significant difference.

**Table 3:** A summary of the median survival time of pleurectomy/decortication vs. extrapleural pneumonectomy for patients with malignant pleural mesothelioma.

Study	P/D MST (Month)	Number of Patients Undergoing P/D	EPP MST (Month)	Number of Patients Undergoing EPP
Branscheid 1991 [21]	10.5	82	9.5	76
Aziz 2002 [22]	14	47	13	64
de Vries 2003 [25]	9	29	12	15
Luckraz 2010 [26]	10.1	90	10.3	49
Rena 2012 [24]	25	37	20	40
Bedirhan 2013 [18]	15	20	17	31
Bovolato 2014 [23]	20.5	202	18.8	301
Infrante 2016 [20]	13	14	19	91

Pleurectomy/decortication: P/D, Extrapleural pneumonectomy: EPP, median survival time: MST.

**Table 4:** Summary of changes in the postoperative pulmonary function.

Disease	Surgical Procedure	Number of Pateints	Preoperative Status	Postoperative Status	Referrence
Chronic empyema	P/D	50	FEV_1.0_%: 61.4 ± 11.6, FVC%: 60.8 ± 11.8	FEV_1.0_%: 78.9 ± 13.3 *, FVC%: 77.4 ± 14.1 *	[1]
Chronic empyema	P/D	26	FEV_1.0_%: 50.0 ± 15.5, VC%: 62.3 ± 13.8	FEV_1.0_%: 69.2 ± 12.7 *, VC%: 79.8 ± 12.9 *	[2]
MPM	P/D	16	FEV_1.0_%: 60.2 ± 10.3, FVC%: 54.7 ± 9.9	FEV_1.0_%: 73.6 ± 11.4 *, FVC%: 68.9 ± 9.1 *	[31]
MPM	extended P/D	36	PS:0 FEV_1.0_%: 84.1 ± 16.9, FVC%: 80.2 ± 4.12 PS:1 FEV_1.0_%: 65.6 ± 14.9, FVC%: 61.7 ± 14.0	PS:0 FEV_1.0_%: 72.4 ± 17.3 *, FVC%: 67.1 ± 3.7 * PS:1,2 FEV_1.0_%: 72.8 ± 17.5, FVC%: 64.8 ± 14.4	[32]
MPM	P/D	16	FEV1.0: 2.65 ± 0.65 *L*, FVC: 3.53 ± 0.91 *L*	FEV1.0: 2.00 ± 0.44 L *, FVC: 2.51 ± 0.65 *L* *	[33]
MPM	P/D	23	median FEV_1.0_%: 87.1%, median FVC%: 86.5%	median FEV_1.0_%: 70%, median FVC%: 68.2%	[17]
MPM	EPP	25	median FEV_1.0_%: 78.0%, median FVC%: 76.8%	median FEV_1.0_%: 49.3 *, median VC%: 48.0% *	[17]

P/D: pleurectomy decortication, FEV1.0: forced expiratory volume in one second, FVC: forced vital capacity, VC: vital capacity, PS: performance status, EPP: extrapleural pneumonectomy, *: significant difference.
